# Impact of Particulate Matter on Hospitalizations for Respiratory Diseases and Related Economic Losses in Wuhan, China

**DOI:** 10.3389/fpubh.2022.797296

**Published:** 2022-05-25

**Authors:** Guiyu Qin, Xuyan Wang, Tong Wang, Dewei Nie, Yanbing Li, Yan Liu, Haoyu Wen, Lihong Huang, Chuanhua Yu

**Affiliations:** ^1^Department of Epidemiology and Health Statistics, School of Public Health, Wuhan University, Wuhan, China; ^2^Union Hospital, Tongji Medical College, Huazhong University of Science and Technology, Wuhan, China; ^3^The Jockey Club School of Public Health and Primary Care, Faculty of Medicine, The Chinese University of Hong Kong, Hong Kong, Hong Kong SAR, China; ^4^Department of Epidemiology and Biostatistics, Institute of Basic Medical Sciences Chinese Academy of Medical Sciences, School of Basic Medicine of Peking Union Medical College, Beijing, China; ^5^Center of Environmental and Health Sciences, Chinese Academy of Medical Sciences, Peking Union Medical College, Beijing, China; ^6^Global Health Institute, Wuhan University, Wuhan, China

**Keywords:** ambient particulate matter, respiratory diseases, hospitalization, economic loss, generalized additive model

## Abstract

**Background:**

Prior studies have reported the effects of particulate matter (PM) on respiratory disease (RD) hospitalizations, but few have quantified PM-related economic loss in the central region of China. This investigation aimed to assess the impacts of PM pollution on the risk burden and economic loss of patients admitted with RD.

**Methods:**

Daily cases of RD admitted to the hospital from 1 January 2015 to 31 December 2020 were collected from two class-A tertiary hospitals in Wuhan, China. Time series analysis incorporated with a generalized additive model (GAM) was adopted to assess the impacts of fine particulate matter (PM_2.5_) and inhalable particulate matter (PM_10_) exposures on patients hospitalized with RD. Stratified analyses were performed to investigate underlying effect modification of RD risk by sex, age, and season. The cost of illness (COI) approach was applied to evaluate the related economic losses caused by PM.

**Results:**

A total of 51,676 inpatients with a primary diagnosis of RD were included for the analysis. PM_2.5_ and PM_10_ exposures were associated with increased risks of hospitalizations for RD. Subgroup analysis demonstrated that men and children in the 0–14 years age group were more vulnerable to PM, and the adverse effects were promoted by low temperature in the cold season. A 152.4 million China Yuan (CNY) economic loss could be avoided if concentrations of PM_2.5_ and PM_10_ declined to 10 and 20 μg/m^3^, respectively.

**Conclusions:**

PM_2.5_ and PM_10_ concentrations were positively associated with RD hospitalization. Men and children were more vulnerable to PM. Effective air pollution control measures can reduce hospitalizations significantly and save economic loss substantially.

## Introduction

According to the Global Burden of Disease (GBD) Study 2019, particulate matter (PM) was one of the largest increased risk exposures worldwide between 2010 and 2019, the percentage of disability-adjusted life-years (DALYs) attributable to PM increased from 2.7% in 1990 to 4.7% in 2019 (1). PM pollution contributed to approximately global 2.49 million deaths and 83 million DALYs in 2017 (2). A province-level disease burden study in China reported that PM was one of the top four risk factors for a number of deaths and accounted for more than 5% of DALYs in 2017 (3).

PM can cause damage to the respiratory system by absorbing toxic metals, carcinogens, and pathogenic bacteria (4); on the other hand, PM deposited in the lung can lead to lung damage by mediating inflammatory response and oxidative stress (5, 6). Population-based epidemiological evidence suggests that PM was associated with respiratory disease (RD) morbidity and mortality (7, 8). Fine particulate matter (PM_2.5_) and inhalable particulate matter (PM_10_) were affirmed to be associated with increased emergency (9, 10) or hospital admissions (11, 12) for asthma (4, 11), pneumonia (13), chronic obstructive pulmonary disease (COPD) (11, 14), and bronchiectasis (11). Some studies also demonstrated the effect of PM on mortality in COPD (14), pneumonia (15), and lung cancer (15, 16). PM was the second-highest risk factor for respiratory tract cancer (tracheal, bronchus, and lung cancers) in 2019, contributing to 15.1% of deaths for these cancers (16). In the past few decades, China has experienced dramatic increases in industrialization and urbanization, accompanied by deterioration of air quality and population health. A study covering 338 Chinese cities revealed that PM_2.5_ caused 1.35 million all-cause premature mortalities, which were equivalent to 17.2% of reported deaths in China in 2017 (17).

Air pollution caused a heavy disease burden, at the same time the economic loss could not be ignored. Air pollution resulted in illness and in turn reduced productivity, decreased working hours and labor supply, and lost welfare (18, 19). An economic study forecasted that the global economic costs of outdoor air pollution gradually increased to 1% of global gross domestic product (GDP) by 2060, with the highest GDP losses in China (20), which mainly reaches to relatively high pollution-related health expenditures and aging population. In China, air pollutants caused economic loss attributable to hospital admission, and premature mortality was 2,065.54 billion China Yuan (CNY), accounting for 2.5% of the national GDP in 2017 (21). A lot of domestic studies analyzed medical costs or economic loss in the northern area like Shanxi (22) and Hebei (23) or economically developed regions like Beijing (24, 25) and the Pearl River Delta (26, 27). However, few studies focused on the central region. Wuhan is the only sub-provincial city in central China. The objective of this study is to provide the risk assessment for RD hospitalization followed by the economic loss evaluation of PM in Wuhan.

## Methods

### Description of the Study Area

Wuhan is the capital of Hubei province. In 2020, Wuhan had a 12.32 million permanent resident population (28). Wuhan is a nationally important industrial base and a comprehensive transportation hub. Vehicle sources and manufacturing emissions were the main air pollution sources in Wuhan (29). Meanwhile, Wuhan is located in the Jianghan Plain, which is one of the largest commercial grain production bases in China (30). Biomass burning also caused heavy pollution in Wuhan (31). The annual average concentration of PM_2.5_ and PM_10_ were 45 and 71 μg/m^3^, respectively in 2019, exceeding the national air quality secondary standard (PM_2.5_: 35 μg/m^3^; PM_10_: 70 μg/m^3^).

### Data Collection

#### Records of Hospitalization

Hospitalization data were obtained from the hospital information system (HIS) of two class-A tertiary hospitals in Wuhan from 1 January 2015 to 31 December 2020. The data consisted of age, gender, principal disease diagnosis, admission date, discharge date, length of stay (LOS), and hospitalization expenses. The diagnosis of the disease was coded according to the International Classification of Disease Tenth Revision (ICD-10). Hospitalization due to all RDs (ICD-10: J00-J99), COPD (ICD-10: J40–J44), and pneumonia (ICD-10: J12-J18) was analyzed in this study. Hospitalization cost was adjusted according to the consumer price index (CPI) to eliminate the impact of price fluctuations.

#### Daily Air Pollutants and Meteorological Data

In recent years, the air pollutant concentrations obtained from ground monitoring networks have been used in many studies to evaluate the health effects associated with air pollution in China (21). Daily mean concentrations of PM_2.5_ and PM_10_ from 1 January 2015 to 31 December 2020 in Wuhan were collected from the Hubei Environmental Protection Bureau (https://sthjt.hubei.gov.cn/), and contemporary meteorological data, such as daily mean temperature and relative humidity, were obtained from China Meteorological Data Network (http://data.cma.cn/).

### Statistical Analysis

A three-stage analysis was applied to assess the impact of PM_2.5_ and PM_10_ exposures on RD hospitalizations and related economic losses.

#### Time Series Decomposition

In the first stage, we decomposed the time-series data of all hospitalizations for RDs, pneumonia, and COPD to detect potential long-term trends and seasonality. The time series data were split into three components (32):


$$\begin{array}{l} Y_{t}=T_{t}+S_{t}+R_{t},\;t=1,2,\;\ldots ,\;n, \end{array}$$

where *Y* is the number of RD hospitalizations, *T*_*t*_ is the long-term trend, and *S*_*t*_ is seasonality and *R*_*t*_ is residual.

#### Impact on Hospitalization

In the second stage, the Spearman rank correlation was applied to measure the association between air pollutants and meteorological data. A generalized additive model (GAM) with quasi-Poisson regression was used to fit the association between PM and RD hospitalization, adjusting for a set of covariates:


$$\begin{array}{l} \log\;\left(E_{i}\right)=\beta_{i}\;\left(C_{i}\right)+ns\;\left(Time,df\right)+ns\;\left(Mt,df\right)\\ \;+ns\;\left(Rh,df\right)+DOW+Holiday+\;\alpha , \end{array}$$

where *E*_*i*_ is the expected value of the hospitalization count for RD on day *i*; β_*i*_ is the regression coefficient; *C*_*i*_ is mean concentrations of air pollutants on day *i*; *Time* is the days of calendar time on day *i* and *DOW* and *Holiday* are dummy variables that represent the day of the week and a public holiday, respectively (12); *ns* represents a natural spline smoothing function (12); *Mt* is the mean temperature; *Rh* is the relative humidity; and α is the intercept. The degree of freedom (*df*) of each variable was chosen referred to the previous study. We initialized the *df* as 4 *df* /year for *Time*, 3 *df* for *Mt* and *Rh* (33).

We calculated the percent change (PC) of hospitalization count for RD attributable per 10 μg/m^3^ addition of PM:


$$\begin{array}{l} PC=\left[exp\;\left(\beta_{i}\times 10\right)-1\right]\times \;100, \end{array}$$

where β_*i*_ refers to the regression coefficient of air pollutants derived from the GAM analysis.

Given that air pollutant exposure had a significant delayed effect on health, this study examined the effect with different lag structures of single-day lag (from lag0 to lag7), where lag0 corresponds to the current day. In addition, we estimated the association between PM and RD hospitalization count for subgroups stratified by gender (male and female), age (0–14, 15–64, and 65+ years), season (warm and cold season), and specific disease (pneumonia and COPD). The warm season was defined as the month of admission date between April and October, and the cold season was the month of admission date between November and March (34).

#### Economic Losses Analysis

In the third stage, we applied the cost-of-illness (COI) approach to estimate the economic loss due to hospital admissions for RD (21). Attributable number (AN) and attributable fraction (AF) are indicators of attributable risk. AN is the number of RD hospitalizations attributed to air pollution. AF represents the proportion of hospital admission contributed to air pollution in total hospitalization:


$$\begin{array}{l} AF=\sum_{i=0}^{n}\left\{1-\frac{1}{\exp\left[\beta \times \left(C_{i}-C_{0}\right)\right]}\;\right\}, \end{array}$$

where β refers to the regression coefficient obtained from the GAM analysis; *C*_*i*_ is the mean concentration of PM on day *i*; *C*_0_ is the threshold concentration of PM, which was assumed to be 0 in this study (21).


$$\begin{array}{l} AN=AF\times \sum_{j=1}^{n}\left(Pop_{j}\times Pro_{j}\right), \end{array}$$

where *Pop*_*j*_ represents the permanent population of Wuhan, which was 10.61, 10.77, 10.89, 11.08, 12.21, and 12.45 million in 2015, 2016, 2017, 2018, 2019 and 2020, respectively. *Pro*_*j*_ is the hospitalization rate of RD. We used the annual RD hospitalization rate of China in 2017 to replace it due to data unavailability, which was 810.22 per 10^5^ population (21).

The economic loss attributable to RD hospitalization related to PM was estimated with the COI method:


$$\begin{array}{l} ECO_{loss}=COST_{mean}+Day_{mean}\times PGDP_{day}\;\\ \;TECO_{loss}=AN\times ECO_{loss}, \end{array}$$

where *ECO*_*loss*_ is the average economic loss for RD hospitalization for each case; *COST*_*mean*_ is the average hospitalization cost for RD of each case; *Day*_*mean*_ is the average LOS for inpatients of RD; *PGDP*_*day*_ is the GDP per capita per day in Wuhan. *TECO*_*loss*_ is the total economic loss for RD hospitalization attributable to PM.

#### Sensitivity Analysis

To test the robustness of the model, we performed a sensitivity analysis by: (1) changing *df* for *Time* (*df* = 3 or *df* = 5) and (2) establishing two-pollutants models (SO_2_, NO_2_, O_3_, CO).

All analyses were performed in R programming language (version 3.6.1, R Foundation for Statistical Computing, Vienna, Austria). The values of *p* < 0.05 were considered to be statistically significant.

## Results

A total of 51,676 RD hospitalization cases were included in this study, of which 60.68% were men. Table 1 presents the descriptive statistics of daily hospitalization counts, air pollutant concentration, and meteorological factors. Daily mean hospitalization counts for all RDs were 23.57. More daily hospitalization counts were observed in men than women. In addition, daily hospitalization counts of the group aged 65+ were higher than that of the 0–14 and the 15–64 age groups.

**Table 1:** Descriptive statistics of daily hospitalization counts of RD, air pollutant concentration, and meteorological factors in Wuhan, China, 2015–2020.

**Variable**	**$\bar{X}$ s**	**Min**	**P_**25**_**	**P_**50**_**	**P_**75**_**	**Max**
**Daily hospitalization counts**						
All RD	23.57, 12.60	0	15	23	32	77
Pneumonia	5.48, 3.94	0	3	5	8	23
COPD	5.68, 3.68	0	3	5	8	30
Male	14.31, 8.00	0	9	14	19	49
Female	9.27, 5.59	0	5	9	13	39
0–14 years	4.34, 3.30	0	2	4	6	21
15–64 years	8.76, 5.43	0	5	8	12	32
65+ years	10.48, 6.22	0	6	10	14	53
Warm season	23.52, 11.17	0	15	23	31	67
Cold season	23.65, 14.39	0	13	23	34	77
**Air pollutants (μg/m** ^**3**^ **)**
PM_2.5_	51.05, 34.50	4	27	43	64	281
PM_10_	81.86, 47.40	3	48	73	107	618
**Meteorological factors**						
Mean temperature (°C)	17.25, 9.20	−3.8	9.0	18.0	25.1	33.9
Relative humidity (%)	79.26, 10.37	41.3	73.0	80.5	87.2	100.0

*Min, minimum; P_25_, the 25th percentiles; P_50_, the 50th percentiles; P_75_, the 75th percentiles; Max, maximum*.

**Figure 1:**
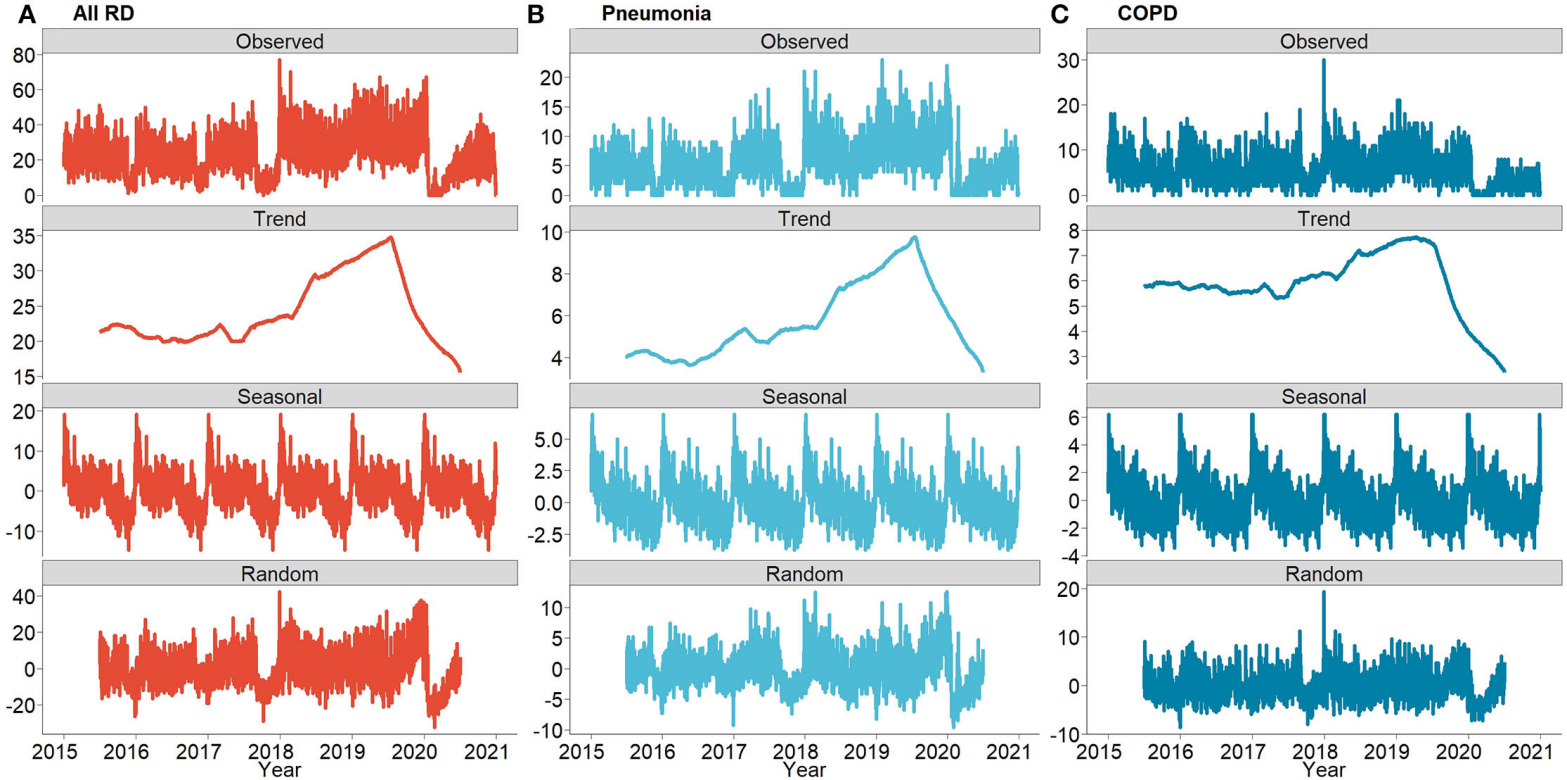
The decomposed distributions for the daily number of hospitalizations due to all RDs, Pneumonia and COPD in Wuhan, China from 2015 to 2020: **(A)** daily number of hospitalizations of all RDs, **(B)** daily number of hospitalizations of Pneumonia, and **(C)** daily number of hospitalizations of COPD.

Figure 1 shows the result of the time series decomposition analysis of the hospitalization count of all RDs, pneumonia, and COPD from 2015 to 2020. The daily hospitalization counts showed an increasing trend between 2015 and 2019 but reduced notably in 2020. The obvious seasonal fluctuations existed in daily hospitalization counts, which were higher in spring and winter than that in summer and autumn.

**Figure 2:**
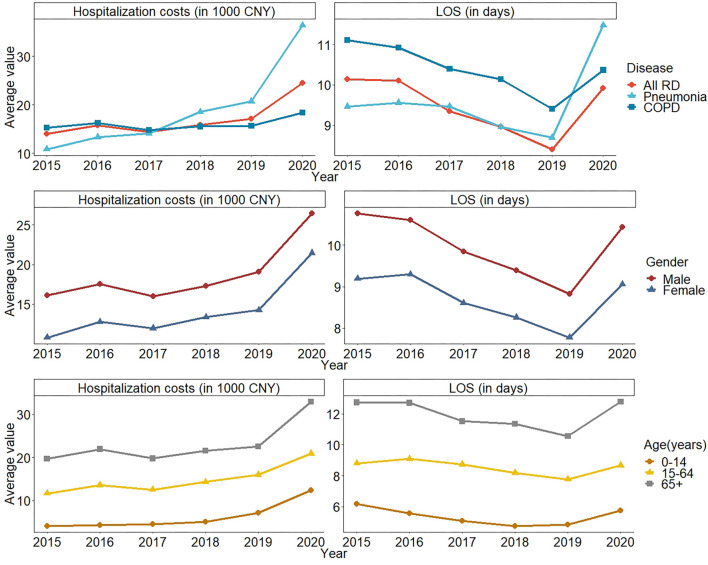
The trend of average hospitalization costs and LOS due to RD in Wuhan, China from 2015–2020 by disease, gender, and age group.

The median of all RD hospitalization costs between 2015 and 2020 was 8,938.16 CNY, and the average LOS of all RDs was 9.33 days. More details are shown in Supplementary Tables S1, S2. Figure 2 illustrates the trend of average hospitalization costs and LOS by disease, gender, and age group from 2015 to 2020. The average hospitalization cost of patients with all RDs and COPD showed a fluctuating trend between 2015 and 2019, then increased in 2020. While the average hospitalization cost of patients with pneumonia showed an increasing trend, exceeding that of all patients with RDs and COPD, and then increased dramatically in 2020. The hospitalization cost of male patients with RD was higher than that of female patients. The age group of 65+ years old had the highest hospitalization cost in all age groups. The average LOS of patients with RD showed a decreasing trend from 10.14 days in 2015 to 8.41 days in 2019 and increased to 9.92 days in 2020. The LOS of patients with COPD was relatively consistent with the trend of all RDs, whereas the LOS of patients with pneumonia increased markedly in 2020 and exceeded all RDs and COPD.

**Figure 3:**
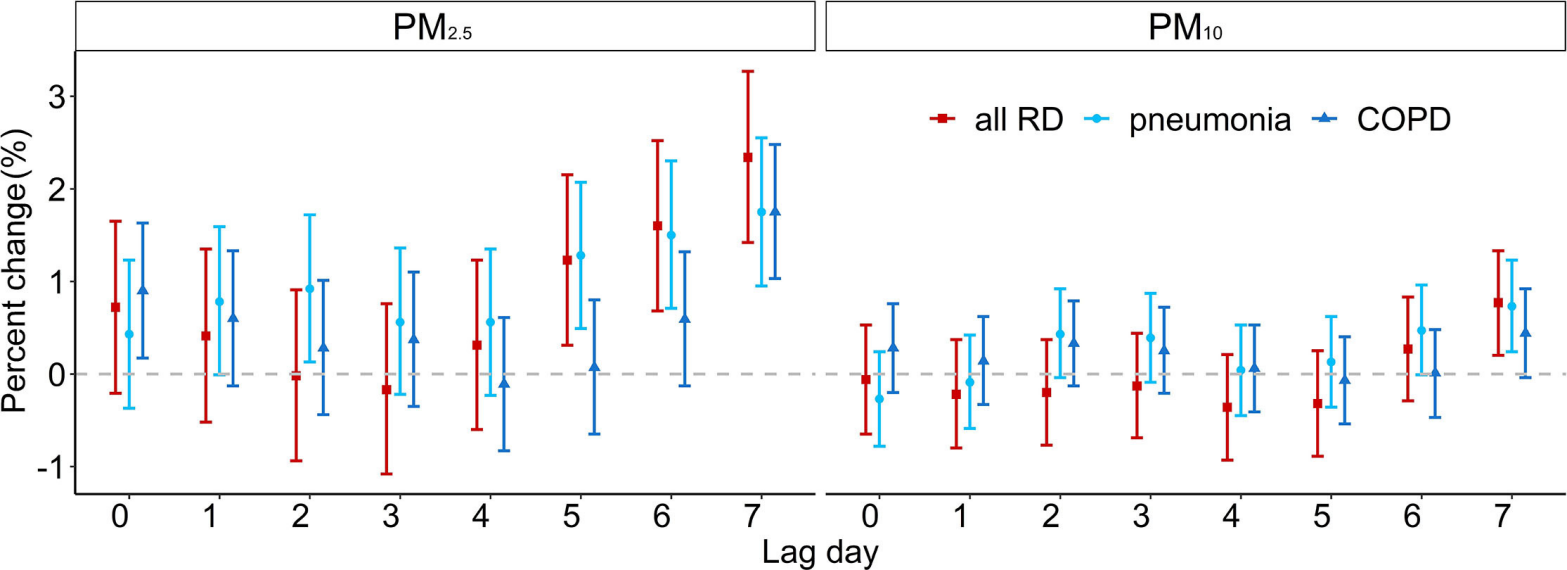
The estimated PC of RD hospitalizations per 10 μg/m^3^ increase in PM_2.5_ and PM_10_ concentrations.

The Spearman rank correlation results are shown in Supplementary Table S3. Briefly, we observed positive correlations between PM_2.5_, PM_10_, SO_2_, NO_2_, and CO but a negative correlation between these pollutants and O_3_. The results of GAM are presented in Figure 3. When PM_2.5_ concentration increased by 10 μg/m^3^, the number of hospitalizations for all RDs increased by 1.23% (95% CI: 0.31, 2.15), 1.60% (0.68, 2.52), and 2.34% (1.42, 3.27) on lag5, lag6, and lag7, respectively. The number of pneumonia hospitalizations significantly increased on lag5, lag6, and lag7, by 1.28% (0.49, 2.07), 1.50% (0.71, 2.30), and 1.75% (0.95, 2.55), respectively. Although no statistical significance was found, we observed a negative association between increasing PM_2.5_ concentration and the number of all RD and pneumonia hospitalizations on lag0–lag4. PM_2.5_ increased COPD hospitalization on lag0 and lag7, by 0.9% (0.17, 1.63) and 1.75% (1.03, 2.48), respectively. Every 10 μg/m^3^ increase of PM_10_ concentration only caused hospitalization increase for all RDs and pneumonia on lag7; the PC was 0.77% (0.20, 1.33) and 0.73% (0.24, 1.23), respectively. Similarly, the increase of PM_10_ showed a decreased effect in the number of hospitalizations on lag0. Also, PM_10_ had no significant effect on COPD.

**Figure 4:**
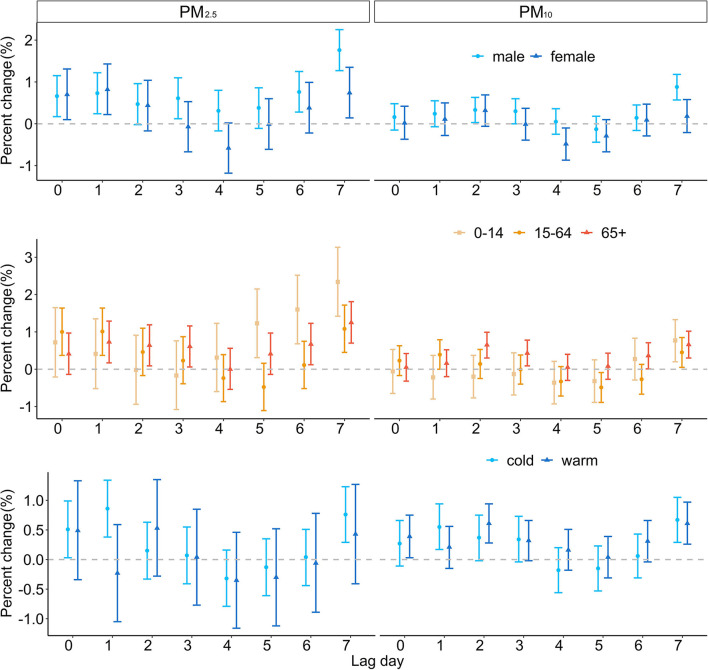
The estimated PC of RD hospitalizations per 10 μg/m^3^ increase in PM_2.5_ and PM_10_ concentrations by gender, age group, and season.

Figure 4 shows the PC of hospitalizations attributable to the increase in PM_2.5_ and PM_10_ concentrations at different lag days by gender, age group, and season. The 10 μg/m^3^ increment in PM_2.5_ caused distinct hospitalization count rise on lag0, lag1, lag3, lag6, and lag7 for men and on lag0, lag1, and lag7 for women; both highest increases occurred on lag7 with 1.76% (1.27, 2.25) and 0.74% (0.14, 1.35). The effect of PM_10_ concentration increase was demonstrated on lag7 with 0.88% (0.57, 1.18) rise for men, and PM_10_ had no significant effect on women. In age-specific analyses, we found that increasing PM_2.5_ and PM_10_ concentration had the greatest effect on the 0–14-year age group, which resulted in 2.34% (1.42, 3.27) and 0.77% (0.20, 1.33) hospitalization increase on lag7. For the 15–64-year age group, the greatest effect associated with PM_2.5_ exposure on the number of hospitalizations appeared on lag7 with 1.08% (0.45, 1.72). For the age group of 65+, the highest PC of hospitalization with a 10 μg/m^3^ increment in PM_2.5_ was found on lag7 with 1.25% (0.70, 1.81). The effects of PM_2.5_ and PM_10_ concentration rise in the cold season were greater than those in the warm season. The largest effect of PM_2.5_ in the cold season was observed on lag7 with 0.76% (0.29, 1.23). The results of disease-specific analysis (pneumonia and COPD) are exhibited in Supplementary Tables S4, S5. On the whole, under the influence of the PM concentration increase, the number of hospitalizations of subgroup showed a trend of first rise, then decline and rise again, which was especially obvious in the influence of PM_2.5_ on women and on the 0–14-year age group.

The result of sensitivity analyses is shown in Supplementary Table S6. After changing *df* for *Time* and establishing two-pollutant models, the effect of PM on hospitalizations remained stable, suggesting that the main model is stable and meaningful.

The lag days with the greatest impact of PM on RD hospitalization were selected for attribution analysis; the results of the RD hospitalization count and economic losses due to PM_2.5_ and PM_10_ are demonstrated in Table 2. The number of RD hospitalizations attributable to PM_2.5_ and PM_10_ was 59.40 thousand and 32.60 thousand, respectively. The economic losses attributed to PM_2.5_ and PM_10_ were 1,304.92 million CNY and 716.29 million CNY. Higher hospitalization count and economic losses were found in men and 65+ year-old age group.

**Table 2:** The number of RD hospitalizations and economic losses attributable to PM_2.5_ and PM_10_ in Wuhan, China, from 2015 to 2020.

**Variable**	**Attributable number of hospitalizations (in thousand)**		**Attributable economic losses (CNY, in million)**	
	**PM_**2.5**_**	**PM_**10**_**	**PM_**2.5**_**	**PM_**10**_**
All RD	59.40	32.60	1304.92	716.29
Pneumonia	13.81	7.58	303.45	166.57
COPD	14.30	7.85	314.21	172.48
Male	36.04	19.78	791.83	434.65
Female	23.35	12.82	513.09	281.65
0–14 years	10.95	6.01	240.47	132.00
15–64 years	22.06	12.11	484.61	266.01
65+ years	26.39	14.49	579.84	318.28

**Figure 5:**
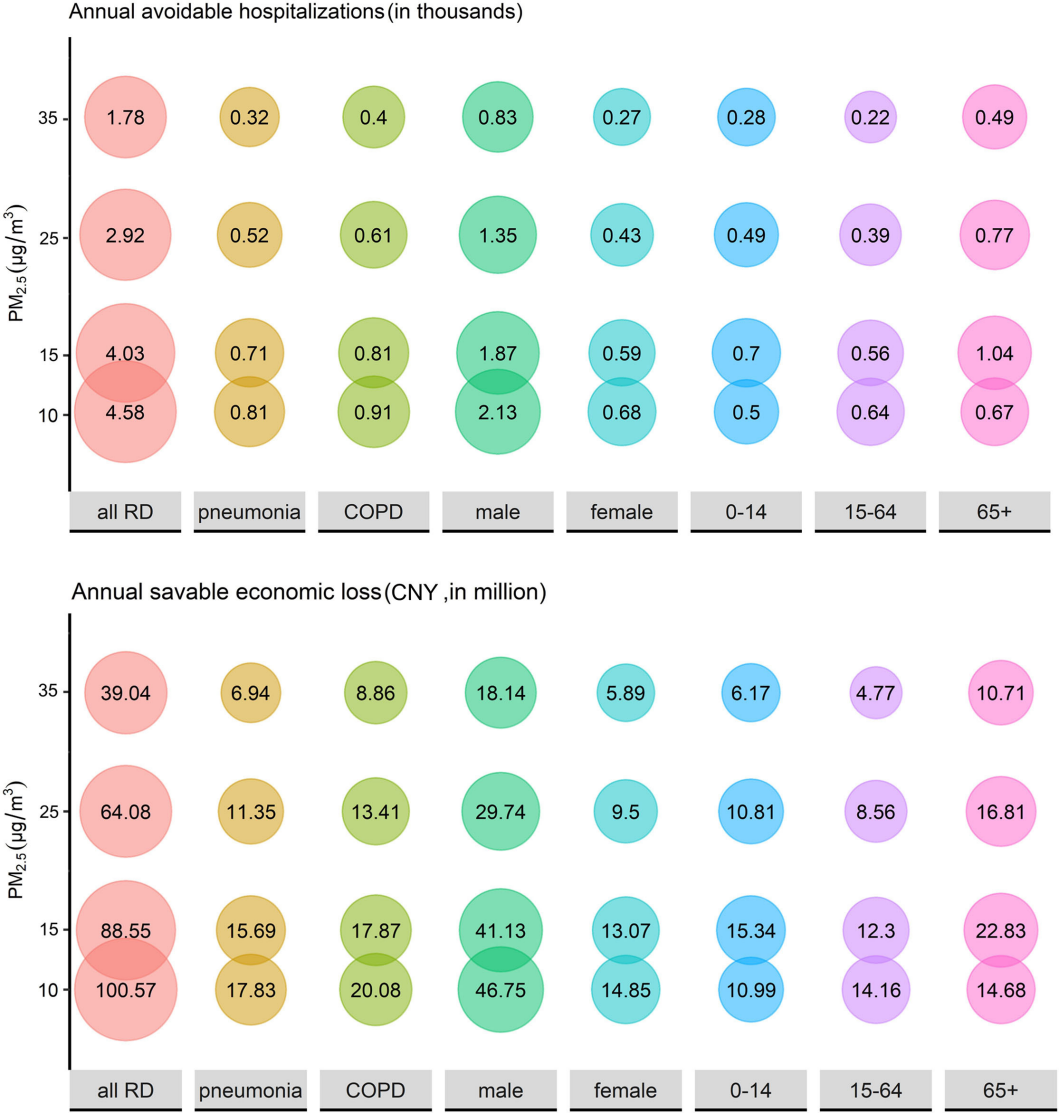
The estimated annual avoidable hospitalizations and savable economic losses if the historical concentration of PM_2.5_ could be maintained at relatively low levels.

**Figure 6:**
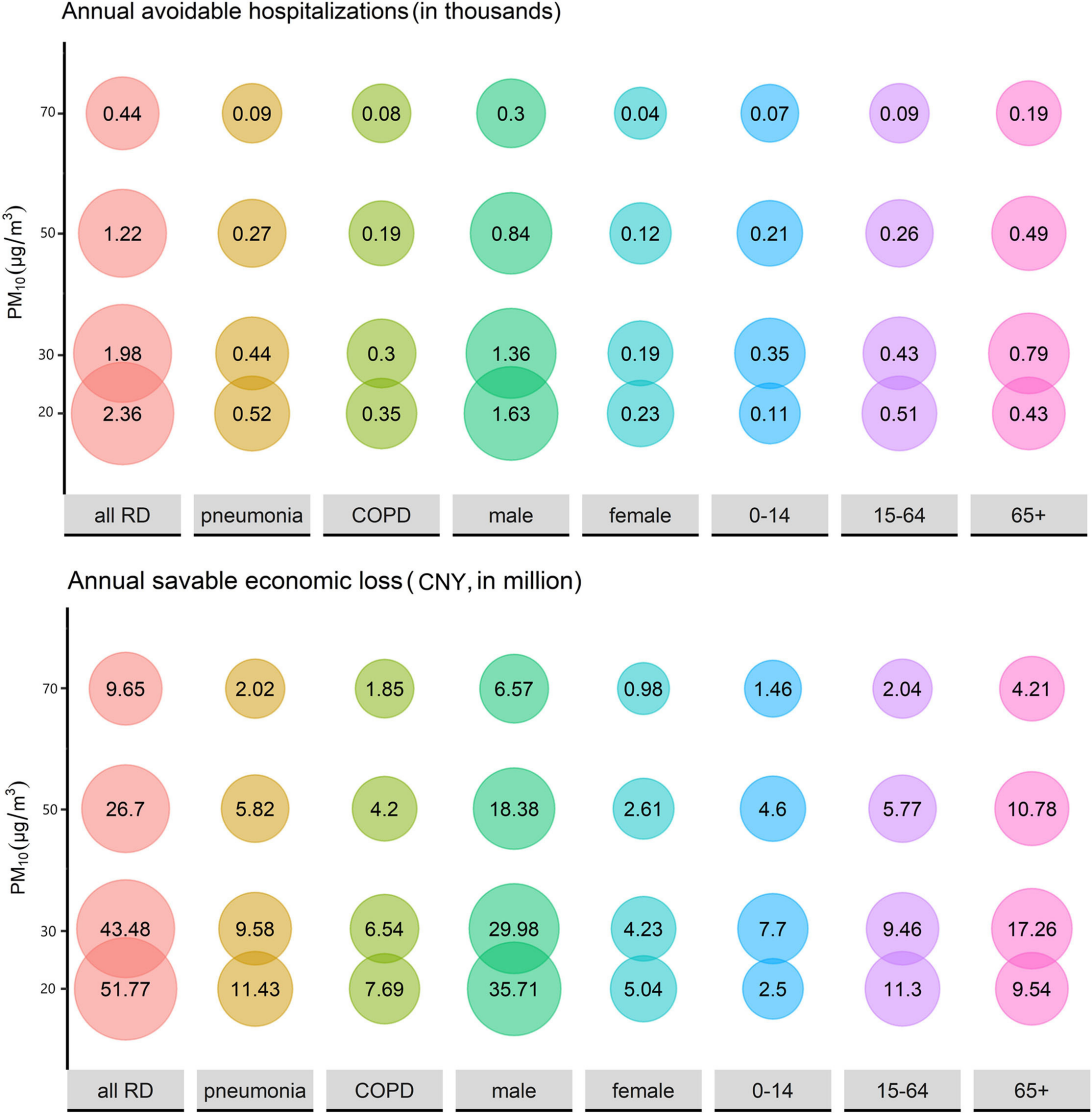
The estimated annual avoidable hospitalizations and savable economic losses if the historical concentration of PM_10_ could be maintained at relatively low levels.

In the WHO 2020 global air quality guidelines, 4 interim target recommendations were proposed for annual concentrations of PM_2.5_ (35, 25, 15, and 10 μg/m^3^) and PM_10_ (70, 50, 30, and 20 μg/m^3^). Assuming that the concentrations of PM_2.5_ and PM_10_ from 2015 to 2020 can reach the WHO guidelines, the annual avoidable hospitalizations and the savable economic losses are displayed in Figures 5, 6. If the annual concentration of PM_2.5_ could reach 10 μg/m^3^, 4,580 hospitalizations and 100.57 million CNY economic losses could be avoided. If the annual concentration of PM_2.5_ could reach 20 μg/m^3^, 2,360 hospitalizations could be avoided for all RDs, and the corresponding cost reduction could be 51.77 million CNY.

## Discussion

In this research, we used comprehensive data to examine the RD hospitalization risk attributable to PM exposure and evaluate the accompanying economic losses. PM_2.5_ and PM_10_ were demonstrated to increase hospitalization for RD, and this influence had a delay effect. Men and 0–14-year-old age group were most vulnerable. PM caused huge RD hospitalization costs; if the concentration of PM_2.5_ and PM_10_ could reach the guideline values recommended by WHO, enormous economic losses could be avoided.

From 2015 to 2019, hospitalizations for all RDs, pneumonia, and COPD showed a fluctuating upward trend, which is consistent with previous findings. In 2020, the number of hospitalizations decreased notably. In early 2020, the global outbreak of coronavirus disease 2019 (COVID-19) pandemic extremely affected people's behavior in work, outdoor activities, and medical treatment. Fears of COVID-19 and tightening hospital admission standards lead to a significant reduction in hospital admissions in 2020. The LOS of discharged patients is an important indicator of hospital performance evaluation (35). In this study, LOS showed a downward trend between 2015 and 2019, which may be related to the hospital actively improving the level of diagnosis and treatment and strengthening the management of hospitalization (36). The influencing factors of hospitalization cost mainly include the LOS, the age of the patient, complications, surgery or not, and intensive care vs. no intensive care (37). In this study, LOS decreased between 2015 and 2019, whereas the hospitalization cost increased at the same time. The increase in the elderly population and the aggravation of the disease may be one of the reasons. In 2020, the LOS and the hospitalization costs increased markedly, and pneumonia exceeded all RDs and COPD, which might have resulted from the COVID-19 pandemic.

The risk estimated in this study was generally higher than those observed in previous studies, especially for PM_2.5_. For every 10 μg/m^3^ increase of PM_2.5_ and PM_10_, we estimated a 2.34% and 0.77% increase in all RD hospitalizations. A study in Nanjing city concluded that every 10 μg/m^3^ increase of PM_2.5_ and PM_10_ concentrations was associated with 0.36% and 0.33% increase in hospital outpatient visits for RD (38). A Chinese national-level study covered 26 cites and concluded that a 10 μg/m^3^ increase of PM_2.5_ was associated with 0.26% increase in RD hospitalization (39). However, studies in New Mexico (40) and Arkansas (41) found no association between air pollution and emergency room visit for RD. PM concentrations and compositions varied substantially over geographical regions in China (42). The difference in the composition of PM, weather conditions, age structure, and population susceptibility may be responsible for the different associations.

Meanwhile, we also found a diverse delay effect model. A study in Nanjing demonstrated the acute response of RD hospital outpatients to PM exposure (38). In this case, the highest effect of PM_2.5_ and PM_10_ appeared on the current day (lag0). In this research, PM decreased RD hospitalization on lag0, and the highest risk appeared on lag7. This study is based on hospitalization date rather than the time of symptoms onset, and the gap may explain the diverse highest risk lag pattern. Although not statistically significant, PM_10_ exhibited a “protective effect” on pneumonia and COPD on lag0, and decreased hospitalizations of all RDs and pneumonia. The “protective effect” may result from the conscious behavior of the study population. The proportion of 65+ year age group was 44.43% in this study. Elderly people with underlying diseases may be more cautious about air pollution. When PM concentrations increase, they may choose to reduce outdoor activities during the day, which may lead to a decrease in hospitalizations on the days of pollution and even in the first 3 days (43). In subgroup analysis, the PC of hospitalization showed a trend of increase, then decrease and increase again, especially for women and 0–14 age groups. These two groups may have lower immunity but less attention to air quality in their daily activities (44). When the PM concentration increases significantly, the exposure on the current day/lag0 has acute effects, such as asthma and pneumonia, and has the greatest impact on chronic RDs, such as COPD, after a lag of 5–7 days.

In this study, we found that the estimated risk of PM_2.5_ was higher than PM_10_ on all lag days. PM_10_ is different from PM_2.5_ in sources, composition, and lung deposition patterns. PM_2.5_ can reach the bronchioles and deposit in the alveoli (13). In addition, PM_2.5_ has a larger surface area than PM_10_, so it can absorb more toxic substances per unit mass (13, 45). For specific diseases, PM_2.5_ exposure had a higher effect on pneumonia (2.11%) than COPD (1.90%), and PM_10_ only had an effect on pneumonia hospitalizations (0.82%). Pneumonia is an acute inflammation of the lower respiratory tract (46). PM caused emergency room visits for pneumonia, and hospitalization increase had been confirmed in the previous study (13, 47). COPD is a chronic RD, and acute exacerbations were the main cause of medical visits and hospitalizations for COPD (48). PM can damage the airway epithelium and can weaken the immune system by oxidative stress and then cause exacerbation of COPD (49).

The subgroup analysis demonstrated that men had a higher increase in PC of hospital admissions for RD than women. A possible explanation could be that men were more inclined to smoking and drinking and that behavioral factors had synergistic effects with air environmental factors (50). A study in Taiwan (51) also indicated that men were more sensitive to PM_10_ exposure. Age differences also existed. In our study, 65+ year age group had the longest LOS and the highest hospitalization cost, which may result in their weakened immune function. However, the 0–14-year age group had a greater increase of RD hospitalization than the older group. Children's respiratory systems are not fully developed, and they are more sensitive to pollutants than middle-aged and elderly people. Moreover, children's outdoor activities increase their exposure to outdoor air pollutants (44). PM had greater effect on RD hospitalization increase in cold season than in warm season, which was consistent with previous studies (41, 52).

Previous studies have found that PM is a risk factor for respiratory infection by carrying microorganisms and affecting body's immunity. The association between PM and COVID-19 pandemic drew attention. PM_2.5_ accelerated COVID-19 spread and its lethality (53), and significantly positive associations were observed in PM with newly COVID-19 confirmed cases (54). Meanwhile, the pandemic may have a different influence on other RDs such as COPD and asthma. Lockdown had a significant impact on the environment and air quality due to reduced industrial activity and traffic, which decreased PM concentration (55). Quarantines and wearing masks reduced PM exposure (56). Taking these factors into consideration, the increase of PM concentrations still leads to an addition in hospitalizations overall, further illustrating the significant impact of PM on RDs and the need for air quality intervention.

PM causes not only RD hospitalization rise but also substantial economic loss. According to our results, PM_2.5_ and PM_10_ exposure led to 92,000 hospitalizations and 2,021.21 million CNY economic loss from 2015 to 2020 in Wuhan. From another perspective, the economic benefits of air pollution control are also considerable. An economic modeling study in Beijing concluded that an incremental monetary benefit from cardiovascular disease decline can offset over two-thirds of the air pollution-control spending if the PM_2.5_ concentration can be reduced to 35 μg/m^3^ and offsets the total spending if the PM_2.5_ concentration can be reduced to 15 μg/m^3^ (24). The same significant effect can be achieved in RDs. A value assessment study calculated that avoided economic loss for RD mortality was 103.5 million dollars when PM_2.5_ dropped to 10 μg/m^3^ in 2017, accounting for 7.31% of all-cause deaths (57). In our study, when the concentration of PM_2.5_ dropped to 10 μg/m^3^, the annual avoidable hospitalization and the annual savable economic losses were 4,580 and 100.57 million CNY, respectively, equating to a 7.71% reduction in both hospital admissions and financial losses. In the same way, when the PM_10_ concentration reached 20 μg/m^3^, the annual avoidable hospitalization and economic losses were 2,360 and 51.77 million CNY, respectively, reducing for 7.24% hospitalization and 7.23% economic loss. Therefore, air pollution control investment can produce enormous monetary benefits, but effective measures should be taken to control PM pollution.

Nevertheless, our study had several limitations. The main limitation of the present study was the unavailability of data on individual exposure to PM pollution. Using monitoring data to represent individual exposure levels may cause measuring errors and underestimate exposure. Patients' information, such as smoke and case history, was unknown, which limited the ability to identify potentially vulnerable people. On the other hand, a combined effect may exist between PM and other air pollutants. Deeper researches are necessary to explore the independent effect of PM on RD. We used hospitalization data of two hospitals to estimate the situation of Wuhan due to data unavailability, and this leads to a certain lack of representativeness in our results, so more comprehensive data are needed in future research.

## Conclusions

In summary, we assessed RD hospitalization and relevant economic loss contributed to PM during 2015–2020 in Wuhan, China. We observed that PM_2.5_ and PM_10_ concentrations at different lag days were positively associated with hospitalization for all patients with RD, pneumonia, and COPD. Men and children were more vulnerable to PM. Effective air pollution control measures can reduce hospitalizations significantly and save economic loss substantially.

## Data Availability Statement

The raw data supporting the conclusions of this article will be made available by the authors, without undue reservation.

## Author Contributions

CY, XW, and GQ: design of study. GQ, TW, LH, and CY: data collection, analysis, and visualization. XW, GQ, and DN: writing the original manuscript. CY, YLi, YLiu, and HW: reviewing and editing the manuscript. CY: funding acquisition. All authors read and approved the final manuscript.

## Funding

This work was funded by the National Natural Science Foundation of China (Grant No. 81773552) and the National Key Research and Development Program of China (Grant No. 2018YFC1315302).

## Conflict of Interest

The authors declare that the research was conducted in the absence of any commercial or financial relationships that could be construed as a potential conflict of interest.

## Publisher's Note

All claims expressed in this article are solely those of the authors and do not necessarily represent those of their affiliated organizations, or those of the publisher, the editors and the reviewers. Any product that may be evaluated in this article, or claim that may be made by its manufacturer, is not guaranteed or endorsed by the publisher.
