# Supplementary material for: Impact of Particulate Matter on Hospitalizations for Respiratory Diseases and Related Economic Losses in Wuhan, China
Source: Front Public Health. 2022 May 25;10:797296. doi: 10.3389/fpubh.2022.797296 (PMC9174547; doi:10.3389/fpubh.2022.797296)
Supplement: Supplementary file 1 [file Data_Sheet_1.docx]

Supplementary Material

**Supplementary Table 1.** Descriptive statistics of hospitalization costs (CNY) for respiratory diseases in Wuhan, China, 2015–2020.

| Variable | Min | P_25_ | P_50_ | P_75_ | Max |
| --- | --- | --- | --- | --- | --- |
| All RD | 8.00 | 5094.31 | 8938.16 | 17187.49 | 1149007.50 |
| Pneumonia | 136.00 | 4592.96 | 7571.61 | 15974.27 | 1149007.50 |
| COPD | 12.35 | 6700.92 | 9604.43 | 16452.56 | 544375.88 |
| Male | 8.00 | 5452.27 | 9971.36 | 19273.89 | 1034689.50 |
| Female | 8.00 | 4737.43 | 7781.05 | 13955.85 | 1149007.50 |
| 0–14 years | 74.14 | 3320.06 | 4074.80 | 5490.45 | 191798.56 |
| 15–64 years | 16.46 | 5422.42 | 8582.40 | 14595.36 | 958932.06 |
| 65+ years | 8.00 | 7583.83 | 12297.70 | 24793.54 | 1149007.50 |
| Warm season | 16.46 | 5111.92 | 9150.26 | 17520.90 | 1034689.50 |
| Cold season | 8.00 | 5067.38 | 8687.69 | 16625.30 | 1149007.50 |

**Supplementary Table 2.** Descriptive statistics of LOS for respiratory diseases in Wuhan, China, 2015–2020.

| Variable | $\bar{X}\pm S$ | Min | P_25_ | P_50_ | P_75_ | Max |
| --- | --- | --- | --- | --- | --- | --- |
| All RD | 9.33±7.81 | 1 | 5 | 7 | 11 | 350 |
| Pneumonia | 9.35±7.90 | 1 | 5 | 7 | 11 | 180 |
| COPD | 10.33±6.79 | 1 | 6 | 9 | 12 | 130 |
| Male | 9.83±8.37 | 1 | 5 | 8 | 12 | 350 |
| Female | 8.57±6.79 | 1 | 5 | 7 | 10 | 191 |
| 0–14 years | 5.25±2.11 | 1 | 4 | 5 | 6 | 85 |
| 15–64 years | 8.44±7.63 | 1 | 5 | 7 | 10 | 350 |
| 65+ years | 11.76±8.57 | 1 | 7 | 10 | 14 | 191 |
| Warm season | 9.28±7.53 | 1 | 5 | 7 | 11 | 191 |
| Cold season | 9.41±8.20 | 1 | 5 | 7 | 11 | 350 |

**Supplementary Table 3.** The coefficient of the Spearman rank correlation between particulate matter (including PM_2.5_ and PM_10_) and SO_2_, NO_2_, O_3_ and CO in Wuhan, China, 2015–2020.

| Air pollutant | PM_2.5_ | PM_10_ | SO_2_ | NO_2_ | O_3_ | CO |
| --- | --- | --- | --- | --- | --- | --- |
| PM_2.5_ | 1.00 |  |  |  |  |  |
| PM_10_ | 0.86* | 1.00 |  |  |  |  |
| SO_2_ | 0.63* | 0.71* | 1.00 |  |  |  |
| NO_2_ | 0.69* | 0.77* | 0.65* | 1.00 |  |  |
| O_3_ | -0.10* | -0.13* | -0.19* | -0.05 | 1.00 |  |
| CO | 0.73* | 0.59* | 0.47* | 0.59* | -0.19* | 1.00 |

Note；**P*＜0.05

**Supplementary Table 4.** The estimated percent change of pneumonia hospitalizations of per 10 μg/m^3^ increase in PM_2.5_ concentrations at different lag days, by gender, age group and season.

| Variable | Lag0 | Lag1 | Lag2 | Lag3 | Lag4 | Lag5 | Lag6 | Lag7 |
| --- | --- | --- | --- | --- | --- | --- | --- | --- |
| PM_2.5_ |  |  |  |  |  |  |  |  |
| Male | 0.43 (-0.64, 1.52) | 0.43 (-0.65, 1.52) | 0.81 (-0.26, 1.89) | 0.94 (-0.13, 2.01) | 0.61 (-0.45, 1.68) | ***1.34 (0.28, 2.41)*** | ***1.74 (0.68, 2.81)*** | ***1.88 (0.81, 2.95)*** |
| Female | 0.31 (-0.89, 1.53) | 1.06 (-0.15, 2.28) | 0.93 (-0.26, 2.15) | -0.06 (-1.26, 1.14) | 0.36 (-0.82, 1.56) | 1.01 (-0.17, 2.22) | 1.03 (-0.16, 2.24) | ***1.56 (0.37, 2.77)*** |
| 0–14 | -0.05 (-1.42, 1.35) | -0.38 (-1.77, 1.02) | 0.65 (-0.72, 2.04) | 0.28 (-1.08, 1.66) | 1.17 (-0.18, 2.54) | ***1.87 (0.52, 3.24)*** | ***2.14 (0.78, 3.52)*** | ***2.43 (1.06, 3.81)*** |
| 15–64 | 1.28 (-0.25, 2.84) | 0.20 (-1.34, 1.77) | -0.35 (-1.88, 1.21) | -0.89 (-2.42, 0.67) | -0.10 (-1.62, 1.45) | -0.03 (-1.56, 1.52) | 0.89 (-0.64, 2.43) | ***1.65 (0.12, 3.2)*** |
| 65+ | 0.19 (-1.10, 1.49) | ***2.11 (0.82, 3.41)*** | ***2.00 (0.72, 3.29)*** | ***1.71 (0.45, 3.00)*** | 0.40 (-0.86, 1.68) | ***1.51 (0.25, 2.79)*** | ***1.29 (0.03, 2.57)*** | ***1.29 (0.01, 2.58)*** |
| Warm | -1.71 (-3.36, -0.04) | -3.16 (-4.80, -1.50) | -2.09 (-3.71, -0.45) | -1.29 (-2.90, 0.35) | -2.39 (-4.00, -0.75) | -1.09 (-2.71, 0.56) | 0.14 (-1.49, 1.80) | -0.44 (-2.09,1.23) |
| Cold | -1.22 (-2.18, -0.26) | -0.44 (-1.39, 0.51) | -0.57 (-1.52, 0.39) | -1.34 (-2.29, -0.38) | -1.03 (-1.97, -0.08) | -0.52 (-1.46, 0.44) | -0.91 (-1.85, 0.04) | -0.45 (-1.40, 0.51) |
| PM_10_ |  |  |  |  |  |  |  |  |
| Male | -0.10 (-0.78, 0.58) | -0.09 (-0.77, 0.59) | 0.28 (-0.37, 0.93) | 0.42 (-0.22, 1.06) | 0.06 (-0.59, 0.72) | 0.02 (-0.63, 0.68) | 0.57 (-0.08, 1.22) | ***0.84 (0.18, 1.49)*** |
| Female | -0.61 (-1.40, 0.18) | -0.24 (-1.01, 0.54) | 0.55 (-0.18, 1.28) | 0.24 (-0.49, 0.99) | -0.10 (-0.84, 0.66) | 0.10 (-0.65, 0.85) | 0.21 (-0.53, 0.97) | 0.57 (-0.18, 1.33) |
| 0–14 | -0.45 (-1.35, 0.45) | -0.61 (-1.50, 0.30) | -0.21 (-1.07, 0.66) | 0.15 (-0.70, 1.00) | 0.09 (-0.76, 0.94) | 0.25 (-0.59, 1.10) | 0.67 (-0.17, 1.51) | -0.45 (-1.35, 0.45) |
| 15–64 | 0.27 (-0.68, 1.23) | -0.40 (-1.37, 0.58) | -0.25 (-1.20, 0.70) | -0.68 (-1.65, 0.29) | -0.53 (-1.49, 0.44) | -0.55 (-1.51, 0.42) | -0.26 (-1.22, 0.71) | 0.67 (-0.27, 1.62) |
| 65+ | -0.70 (-1.53, 0.15) | 0.37 (-0.42, 1.18) | ***1.30 (0.57, 2.04)*** | ***1.15 (0.41,1.9)*** | 0.24 (-0.54,1.03) | 0.28 (-0.51,1.07) | 0.64 (-0.13,1.43) | ***0.93 (0.14,1.73)*** |
| Warm | -0.40 (-1.14, 0.35) | -0.80(-1.53, -0.05) | 0.03(-0.65, 0.70) | 0.08 (-0.58, 0.75) | -0.53 (-1.23, 0.18) | -0.33 (-1.03, 0.37) | 0.19 (-0.49, 0.87) | 0.12 (-0.58, 0.83) |
| Cold | -0.97 (-1.73, -0.20) | -0.39 (-1.15, 0.38) | -0.10 (-0.86, 0.67) | -0.21 (-0.97, 0.56) | -0.36 (-1.12, 0.40) | -0.39 (-1.14, 0.37) | -0.49 (-1.25, 0.27) | 0.04 (-0.72, 0.81) |

**Supplementary Table 5.** The estimated percent change of COPD hospitalizations of per 10 μg/m^3^ increase in PM_2.5_ and PM_10_ concentrations at different lag days, by gender, age group and season.

| Variable | Lag0 | Lag1 | Lag2 | Lag3 | Lag4 | Lag5 | Lag6 | Lag7 |
| --- | --- | --- | --- | --- | --- | --- | --- | --- |
| PM_2.5_ |  |  |  |  |  |  |  |  |
| Male | 0.52 (-0.35, 1.40) | 0.44 (-0.44, 1.32) | -0.10 (-0.97, 0.78) | 0.23 (-0.64, 1.10) | -0.28 (-1.14, 0.59) | -0.12 (-0.99, 0.76) | 0.42 (-0.44, 1.30) | ***2.26 (1.40, 3.13)*** |
| Female | ***1.80 (0.46, 3.15)*** | 0.74 (-0.60, 2.10) | 0.87 (-0.46, 2.22) | 0.44 (-0.88, 1.79) | 0.01 (-1.31, 1.36) | 0.51 (-0.82, 1.86) | 1.11 (-0.23, 2.46) | 0.68 (-0.66, 2.03) |
| 0–14 | ***7.85 (0.73, 15.47)*** | 1.91 (-4.99, 9.31) | -1.03 (-7.96, 6.42) | -4.20 (-11.21, 3.37) | -0.44 (-7.32, 6.94) | -1.78 (-8.57, 5.51) | 4.57 (-2.27, 11.9) | ***8.56 (1.70, 15.89)*** |
| 15–64 | 1.05 (-0.42, 2.54) | 1.24 (-0.23, 2.74) | 0.67 (-0.79, 2.15) | 0.27 (-1.18, 1.75) | -0.46 (-1.91, 1.01) | -0.23 (-1.69, 1.25) | 1.26 (-0.19, 2.73) | ***3.14 (1.69, 4.60)*** |
| 65+ | 0.78 (-0.07, 1.64) | 0.30 (-0.55, 1.16) | 0.08 (-0.77, 0.93) | 0.40 (-0.44, 1.25) | -0.05 (-0.89, 0.79) | 0.24 (-0.61, 1.09) | 0.40 (-0.45, 1.25) | ***1.27 (0.43, 2.13)*** |
| Warm | -1.36 (-2.97, 0.28) | -1.95 (-3.55, -0.32) | -0.83 (-2.41, 0.78) | -1.17 (-2.75, 0.43) | -2.14 (-3.72, -0.54) | -1.86 (-3.46, -0.24) | -1.85 (-3.46, -0.22) | -1.40 (-3.02, 0.23) |
| Cold | -0.42 (-1.27, 0.44) | -0.74 (-1.59, 0.12) | -1.38 (-2.23, -0.52) | -1.01 (-1.87, -0.15) | -1.42 (-2.27, -0.57) | -1.32 (-2.17, -0.46) | -0.74 (-1.60, 0.11) | 0.45 (-0.40, 1.30) |
| PM_10_ |  |  |  |  |  |  |  |  |
| Male | 0.23 (-0.34, 0.81) | 0.20 (-0.37, 0.78) | 0.25 (-0.31, 0.81) | 0.14 (-0.42, 0.70) | -0.01 (-0.57, 0.56) | -0.19 (-0.76, 0.38) | -0.14 (-0.71, 0.44) | ***0.83 (0.27, 1.40)*** |
| Female | 0.37 (-0.52, 1.27) | -0.21 (-1.11, 0.69) | 0.31 (-0.56, 1.18) | 0.33 (-0.53, 1.19) | -0.01 (-0.88, 0.87) | 0.20 (-0.67, 1.07) | 0.41 (-0.46, 1.29) | -0.44 (-1.36, 0.48) |
| 0–14 | ***6.06 (0, 12.49)*** | -0.50 (-6.39, 5.77) | -1.80 (-7.70, 4.48) | -3.29 (-9.18, 2.97) | -0.68 (-6.57, 5.58) | -1.06 (-6.84, 5.08) | 3.18 (-2.58, 9.27) | ***8.63 (2.77, 14.84)*** |
| 15–64 | -0.29 (-1.26, 0.69) | 0.28 (-0.66, 1.23) | 0.17 (-0.75, 1.09) | 0.22 (-0.69, 1.14) | -0.58 (-1.52, 0.37) | -0.36 (-1.30, 0.58) | -0.38 (-1.33, 0.57) | 0.87 (-0.06, 1.80) |
| 65+ | 0.43 (-0.13, 0.99) | 0.04 (-0.53, 0.60) | 0.33 (-0.21, 0.88) | 0.23 (-0.31, 0.78) | 0.22 (-0.33, 0.77) | 0.05 (-0.50, 0.60) | 0.16 (-0.39, 0.72) | 0.28 (-0.28, 0.85) |
| Warm | -0.40(-1.23,0.43) | -0.09(-0.87,0.70) | 0.61(-0.10,1.31) | 0.56(-0.14,1.27) | -0.31(-1.08,0.46) | -0.05(-0.82,0.72) | 0.14(-0.63,0.91) | 0.38(-0.40,1.17) |
| Cold | -0.22 (-0.90, 0.47) | -0.74 (-1.43, -0.06) | -0.68 (-1.37, 0.01) | -0.60 (-1.28, 0.09) | -0.65 (-1.33, 0.03) | -0.70 (-1.38, -0.02) | -0.72 (-1.39, -0.03) | -0.11 (-0.80, 0.58) |

**Supplementary Table 6.** Results of sensitivity analyses by adjusting for two-pollutants and changing the degree of freedom for the long-term trend and seasonality. Results are shown in percent change (%) per 10 μg/m^3^ increase in PM_2.5_ and PM_10_ concentrations at the best lag day.

| Variable | Main Model | Changing degree of freedom | |  | Adjusting for co-pollutants | | | |
| --- | --- | --- | --- | --- | --- | --- | --- | --- |
|  |  | *df*=3 | *df*=5 |  | +SO_2_ | +NO_2_ | +O_3_ | +CO |
| PM_2.5_ | 2.34 (1.42, 3.27) | 1.17 (1.54, 1.91) | 1.09 (0.71, 1.47) |  | 1.36 (0.98, 1.74) | 1.37 (0.99, 1.75) | 1.35 (0.97,1.74) | 1.37 (0.99,1.75) |
| PM_10_ | 0.77 (0.20, 1.33) | 0.68 (0.44, 0.91) | 0.67 (0.42, 0.92) |  | 0.61 (0.36, 0.85) | 0.61 (0.37, 0.85) | 0.60 (0.36,0.84) | 0.61 (0.37,0.85) |
